# Bibliographical Notices

**Published:** 1842-10-01

**Authors:** 


### Periscope;

OR,

### CIRCUMSPECTIVE REVIEW.

" Ore trahit quodcunque potest, atque addit acervo."

### Notices of some New Works.

### On the Hydropathic Cure of Gout. By *G. Hume Weatherhead,* M.D. Highley, 1842.

Nobody knows better than Dr. Weatherhead that the term "Hydropathic Cure" is a solecism in language—" Water-disease for the Cure of Gout!" The term Hydro-therapeia has been properly applied to it by some continental writers. In one sense, however, hydropathy is a good term, as the remedy is more likely to produce than remove a disease. We have expressed our opinion so recently on the probable effects and fate of this Germano-mania, that we need not repeat it here; but confine ourselves to the subject of gout, inquiring into the doctrine and practice of our author in this painful and frequent malady.

Dr. Weatherhead acknowledges that the cold-water cure of gout is no new practice. We all remember the doctrine of Kinglake—now completely repudiated by every experienced practitioner in England. We are therefore surprized at the following sentence.

"Though much has been already written against refrigerant applications in the acute stage of the gout, and the minds of patients terrified by the imminent dangers of repulsion, no authentic proof of the fact has yet been adduced."

We have seen more than twenty instances where cold applications to gout in the feet, caused most violent cramps and spasms in the stomach, or such determinations to the head as threatened apoplexy. Dr. W. indeed sets up a man of straw, in the shape of warm wool or flannel—a practice which is rarely employed in *acute gout* (which is the state he is speaking of) by modern practitioners. No one denies that spontaneous retrocessions and transpositions of gout take place —and that frequently; but is that a reason why, by cold applications, we should encourage or actually produce the migrations of the disease? Just the reverse. But let that pass for the present. Dr. W. gives us, in a brief manner, his theory of the nature of gout.

"If we carefully attend to the circumstances under which the disease appears, the ailments by which it is preceded, certain of the symptoms with which it is accompanied, and those which terminate the paroxysm, we shall detect one invariable concomitant of them all, capable, if I be not deceived, of determining both the essential nature of the gout, and the cause producing it, and that is——acidity, in some form or other."

Now granting, argumenti causa, that in every case of gout there is acidity, both of stomach and urine, it does not necessarily follow that the acidity is the cause of the gout. For one case presenting gout in combination with acidity, there are 99—nay, 599, where these acidities prevail, without any gout at all. What is the cause of the acidity? Imperfect digestion. Then why should not the indigestion give origin to both the gout and the acidity? This is the doctrine held by nine-tenths of experienced practitioners at the present time.

Dr. W. supports his doctrine on the authority of Van Helmont, Berthollet, Murray, Forbes, &c. but, after all, he comes to the very conclusion which all the world has come to before him.

"The source whence the elements of this acidity are derived will be shown more at large hereafter to be the digestive organs. For the present it merely may be observed, that those whom the gout eventually attacks usually have, for a length of time preceding the seizure, suffered from symptoms of indigestion, specially characterised by *acidity* and flatulence."

Thus then, Dr. W. and ourselves are almost of the same opinion—except that we consider the acidity as the *effect* of indigestion, without perceiving any proof of its being the *cause* of gout.

We need not describe the symptoms of this rich man's malady; but proceed at once to our author's treatment. We shall not argue the question whether the application of cold water to a gouty limb can or cannot cause its migration to another, and perhaps more important part. Dr. W. says it will not. We say it may—let the practitioner take which of these two opinions he likes best.

We perfectly agree with our author in thinking Dr. Kinglake entirely mistaken in his theory of gout—namely, that it was neither more nor less than common inflammation. We coincide with Dr. W. in looking on the inflammation as specific and constitutional, and that, whatever may be the local means employed, there must be constitutional treatment also. The treatment of Priessnitz, advocated by our author is this:—

"If the patient be comparatively strong he provokes general perspiration by enveloping the body in a woollen blanket, and solicits and encourages the discharge from the skin, by administering water as drink as soon as it is fairly established, and to flow in a more especial manner from the parts affected, by wrapping them in the heating bandages. By these means, besides equalizing the circulation, and thus withdrawing the determination of blood to the foot, or where else the disease has its seat, the gouty acrimony is being discharged from the humours by the perspiration. Now, when Priessnitz has continued this process for half an hour or an hour, according to circumstances, he removes the patient, if not too debilitated, either to the cold bath or the douche, or to tepid ablutions simply, if he be much enfeebled. Another advantageous modification is the substitution of perspiring by first wrapping the body in a wet sheet, and then in a blanket; the general application, in fact, to the whole body of the principle on which the heating bandage operates. It is found better to use cold ablutions than the cold bath after the wet sheet. It is necessary to bear in mind that the means of cure require to be moderated, if the patient be unable to leave his bed.

As soon as the patient has undergone the above treatment he is to be rubbed thoroughly dry, to dress quickly, and, *if not incapacitated by pain or weakness*, to take exercise in the open air. The best of all exercises is that of walking, and while doing so the patient is to drink liberally of cold pure spring water."

Now this splashing and ingurgitation of cold water in an attack of acute gout, may do very well for Dr. Weatherhead, the stamina of whose constitution are as strong and unbending as the basaltic pillars of Staffa; but we know, from long experience, personal and otherwise, that, in nine cases out of ten, of confirmed gout, and after the age of 40, this plan would verify the meaning of the word HYDROPATHY, and induce a real, and sometimes fatal "WATER-DISEASE."

The practice of plunging into cold water after warm or vapour baths, as a *preventive* of disease, and in sound health, is a very different thing from the same practice in acute inflammations of internal organs—or of specific inflammations externally, as of gout, erysipelas, &c.

And after all, have we not a much safer, and, we will venture to affirm, a much more effectual mode of dispersing the actual paroxysm of gout than this hydropathic experiment? We are convinced that we have. It is obvious that the Priessnitz plan embraces no internal remedies, except the ingurgitation of cold water which, in many cases, would be injurious, if not fatal. Yet the constitutional treatment of this disease is of infinitely more importance than the topical. The safer and best plan is to clear the primæ viæ by aperients adapted to the age and strength of the patient, but into which a mercurial should always enter. Thus a calomel and colocynth pill should be followed by saline or senna and saline purgatives till the bowels are well cleared, when salines with colchicum should be administered every four or six hours, with minute doses (say half a grain of calomel and a grain or two of James's powder) of mercurial diaphoretics. This is a safe and efficacious procedure, as far as internal means are concerned, and as compared with the hydromania of Priessnitz. As to the topical remedy, we fearlessly aver, from severe personal sufferings and ample experience, that the tepid evaporating lotions are infinitely more efficacious, and beyond all comparison more safe than the cold sheets, blankets, or towels. Whoever tries the somewhat hazardous experiment of putting a gouty foot or hand into cold water, will hardly forget the stunning, benumbing, and indescribable pain which it produces. After this, the re-action is great, and the heat and pain of the parts are ten times more than before the immersion. Now a tepid lotion, composed of vinegar, spirit, and water, produces a soothing effect from the very first moment of its application, and the subsequent evaporating process carries off the caloric and relieves the pain very much better than the cold water, besides being infinitely safer.

Dr. Weatherhead, who is a clear-headed and learned physician, will do well to ponder on the effect which his adhesion to the Priessnitz empiricism (for as far as the original Peasant is concerned, it is empiricism) will have on the propagation of hydropathy. The disciples of that science (for it lays claim to the title) will hail the advent of a regular physician with great exultation. But let them beware. It is along time since Hudibras exclaimed—

> " Ah ! me, what perils do environ
> The man that meddles with *cold* iron !"

But could some of Kinglake's disciples put a foot out of their graves, they would be apt to warn their survivors against *cold* water in gout, as infinitely more dangerous than cold, or even red-hot iron.

---

HASTINGS ; CONSIDERED AS A RESORT FOR INVALIDS, &c. &c. &c. By *James Mackness,* M.D. Physician to the Hastings Dispensary. 8vo. Churchill, 1842.

It is a wonder that HASTINGS has not sooner sent forth an account of its salubrious atmosphere in pulmonary complaints. It certainly is not the worst locality in Europe for invalids—and many, who seek the bright skies of fair Italy, would be wiser to plant themselves for the Winter in the neighbourhood of Pelham Crescent.

Dr. Mackness, as physician to a public dispensary in Hastings, had ample opportunities for observing the comparative frequency of particular maladies, and was struck with the comparative *infrequency* of certain destructive diseases that prove the terror of Englishmen.

From statistical facts it was ascertained " that tubercular consumption was far more rare amongst the inhabitants of Hastings than in other places, while several diseases of a contagious character were either extremely rare or altogether absent." These statistical facts were published in the Gazette and Lancet, but are now expanded into a small volume for more permanent record.

*Soil and Climate.*—The soil of Hastings is composed of immense beds of sand and sand-rock, with calciferous grit, fuller's earth, slaty clay, and shale with iron. There is abundant radiation of heat from the light colour of the surface, while the thirsty nature of the soil absorbs all humidity, allowing but little evaporation, and thus preventing dense and cold land-fogs. The sea-fogs, to which Hastings, in common with other parts of the coast, is liable, deposit no dew. In the Summer season invalids are able to go out in a few hours after a heavy fall of rain. The scenery of the cliffs and downs is very beautiful and exhilarating. The geological character of the soil prevents entirely the formation of malaria.

Hastings and St. Leonards are well sheltered, being protected on the North and East by some of the most elevated land in Sussex—the hill of Fairlight being 541 feet above the sea. On the West, too, Hastings is screened by a continuous line of hill rising from 2 to 300 feet above the Channel, to which the town is open on the South. The old part of the town is still better sheltered than the new.

Our author considers it no small recommendation to Hastings that there is, in its vicinity, a chalybeate as powerful, or more so, than Tunbridge Wells. The iron exists in the form of protoxide, and is held in solution by carbonic acid gas. It contains about 4½ grains in the imperial gallon, with 5 of muriate of lime—1½ sulphate of lime—3 sulphate of soda—4 sulphate of magnesia—4 carbonate magnesia—total 23 grains of solid matters in the gallon. There are also 18½ cubic inches of carbonic acid gas—1½ oxygen—5 azotic gas. Thus, it appears that the Hastings spa is richer in iron by nearly one-half, and in respect to carbonic acid gas, it contains double the quantity of Tunbridge Wells. Half-a-pint, twice a day, is the usual dose of the Hastings chalybeate. This water may prove a useful tonic in certain complaints of debility, unattended with any organic disease; but to the great majority of patients resorting to Hastings, as a Winter residence for pulmonary diseases, the chalybeate will be entirely useless.

In the third chapter our author takes up the subject of vital statistics, and republishes a paper from two periodicals illustrative of that point. It appears that the total number of cases entered on the dispensary books of Hastings for twelve years, was 7741, and the deaths in the Borough, from all causes, were 865. In this category there were 161 deaths from consumption—91 among the inhabitants, and 70 among strangers. There were, however, 64 deaths from other affections of the chest—many of which were probably of a phthisical character. The list shews exceedingly few of those diseases which are of miasmatic origin. There were only seven cases of typhus in the dispensary practice—and six deaths from that disease in the whole of Hastings during 12 years.

"According to the report of the registrar-general, the proportion of deaths arising from typhus fever in the whole kingdom, is about one for every sixteen of those who die from other causes; the mortality at Hastings has been only six cases out of 865 deaths, or only in the proportion of one for every 144, so that this fatal and dreadful malady has been nine times less frequent at Hastings than the usual average in other parts of England."

Upwards of 150 cases of ague have occurred at the dispensary, and the author considers a former state of bad drainage now removed. Cholera did not visit Hastings; but that exemption was participated in by many other watering-places. No death from acute rheumatism has occurred. Longevity is considerable at Hastings. We must pass over the 4th chapter, on CONSUMPTION, as it offers nothing new to the medical reader—nor could it be expected to do so.

The FIFTH chapter treats of those "diseases for which the climate of Hastings is suitable."

This reminds us very much of those chapters by the spa-doctors where the spring is represented as THE cure for almost all diseases. Dr. Mackness, however, is very moderate in this respect. His list embraces only the following items, though some of them are very important ones. "Consumption—chronic bronchitis—asthma—neuralgia—rheumatism—gout—scrofula—indigestion—

—atonic dyspepsia—inflammatory dyspepsia—strumous dyspepsia—diseases of children—diseases of the skin, &c." A short sketch of these complaints, "serving as a guide to the general reader," follows; but with a wise proviso, that "it is not intended to lead patients to become their own medical advisers." Of the sincerity of this proviso we have not the smallest doubt.

Our author agrees with Sir James Clark in the following sentiments.

"Judging from my own observation, I should say that the climate of Hastings is unfavorable in nervous headaches, connected with or entirely dependent upon an irritable condition of the digestive organs, and also in cases where a disposition to apoplexy or epilepsy has been manifested, but it will be understood from what has been already stated respecting the topographical relations of Hastings, that this effect of its climate is chiefly experienced in the lower and more confined parts; nor is such an effect peculiar to this place; it is common, I believe, to all places similarly situated. The class of persons alluded to, if induced to reside for any length of time at Hastings, should avoid the more confined situations below the cliff, and rather seek such quarters as are more open and elevated, yet in some degree protected from the north and east winds."

There are two or three short chapters on locality, dress, exercise, &c. &c., which will prove useful to the invalid, when taking up his winter quarters in Hastings; but which do not require notice in a medical review. Dr. Mackness's little volume will doubtless increase considerably the reputation of this already favourite resort of valetudinarians, and direct thither no small accession of all such visitors as may labour under any of the complaints specified in the *Carte de Maladies.* We beg all such to attend to the hint of our author, viz. "*not to become their own medical advisers.*"

---

The Evolution of Light from the Living Human Subject. By Sir *Henry Marsh,* Bart. M.D., President of the King and Queen's College of Physicians, Dublin, &c. &c. &c. Pp. 59. W. Curry & Co. Dublin, 1842.

The substance of this brochure was read before the College in Dublin, some four years ago, and an abstract of it published in the proceedings of that body. In the present publication Sir Henry has embodied a great deal of research and communications respecting the subject of animal luminescence (if we may use such an expression) as well as that from various inorganic substances. By using the term *phosphorescence,* our author does not pledge himself to prove or even suppose an identity between phosphorus and the luminescence in question.

Sir Henry has collected a great many curious examples of this luminous phenomenon. It has been seen playing in lambent corruscations on the masts and yards of ships, or rendering the ocean a sea of liquid fire. Dr. Lynch, of Galway, witnessed a remarkable example of this phosphorescence, while travelling by night over an extensive peat-bog in Ireland. Cones of intense fire rose in all directions around him, varying from six to thirty feet in diameter, at their base, and rising to 30 feet, more or less, in height. A heavy rain came on, and dispersed these astounding nocturnal fellow-travellers into air—thin air.

The phosphorescence of fishes, when in a state of decomposition, is well known. It ceases when the putrefaction is complete. Equally familiar is the radiation of light from rotten wood, giving rise to strange stories among the young as well as the old inhabitants of rural districts. The luminous exhalations that occasionally rise from burial-grounds, produce still more terror, and have even been pressed into the service of the muse.

"Thy midnight cup is pledged to slaves,
No genial ties enwreath it,
The smiling brow, like light on graves,
Has rank cold hearts beneath it." *Moore.*

Living vegetables, as well as living animals, sometimes exhibit this luminescence. Thus several species of the lichens, giving to damp cellars and mines a curious and brilliant appearance. But it is in the ocean, especially in tropical climates, that the most magnificent scenes of marine phosphorescence are witnessed. Sometimes the surface of the sea exhibits a diffused sheet of light—but most commonly the waves sparkle with intermitting and often vivid scintillations; while the track, or wake of the ship presents a long and illuminated VIA LACTEA, as far as the eye can reach. The smooth and placid waters of the Mediterranean often present these luminescences as the boats cut their way through the deep, or the oars plunge into the glassy surface.

"Flash'd the dipt oar, and sparkling with the stroke,
Around the waves phosphoric brightness broke." *Byron.*

But we must leave the immense mass of facts collected by our author on this subject, in order to come at once to the phenomena that gave rise to Sir Henry's paper originally.

About ten days before the death of a lady, the narrator observed an extraordinary light darting about her face and head, flashing like an aurora borealis. She was in the last stage of consumption, and had had, that day, an attack of suffocation which lasted for an hour, and left her very nervous. The narrator watched this luminous phenomenon for some time, when it disappeared. It gave the face an appearance as though it had been painted white and highly glazed. But the light danced about in a very strange manner. Three nights afterwards the narrator sat up again with the young lady, and again observed the luminescence, although there was no candle in the room, nor any moonlight. The patient's sister came into the room and observed it also. It was again seen the night before death took place, but the light was fainter. Her breath had a peculiar smell, which led the observer to think that decomposition was going on.

Sir H. avers that the narrator was a man of clear head, superior observation, and free from superstition. The next instance fell under our author's own observation. A young lady was in the last stage of phthisis like the other, and had read some account of the phenomenon. She was much interested on the topic, and often conversed with Sir Henry about it. About an hour and a half before her death, her sister and some other friends were "struck by a luminous appearance proceeding from her head in a diagonal direction." She was semi-recumbent and tranquil. "The light was pale as the moon." At first it was thought to be lightning, but this was given over. As the light played round the head of the bed, candles were brought in lest the dying person should observe it. This case is not so satisfactory as the first.

The third instance was in that of a man who died of lingering disease in the South of Ireland. "All the witnesses agree in having seen the light; but many of them came to the conclusion that it was caused by supernatural agency." Dr. Donovan, however, visited the cabin where the man lived for 14 nights, and "on three nights only did he witness anything unusual." Once he perceived a kind of luminous fog—and twice he saw scintillations, like the sparkling phosphorescence of the marine infusoria. He seems certain that there was no imposition. The luminescence in question was not seen on the man's person, but over the head of his bed, on a wall composed of clay and mortar. The luminous fogs appeared to pass in streams through the apartment.

We have only space to allude to one more instance, observed by Dr. W. Stokes. There was a poor woman in the Meath Hospital, labouring under enormous cancer of the breast, from all parts of the ulcerated surface of which, a quantity of luminous fluid constantly distilled. The poor woman remarked that the sore was on fire every night. The light was visible to Dr. Stokes at the distance of 20 feet.

For many ingenious speculations and rational explanations of this curious phenomenon, we must refer to the paper itself, which is clearly an emanation from a classical scholar, experienced physician, and enlightened philosopher.

---

Deformities of the Spine and Chest, successfully treated by Exercise alone; and without Extension, Pressure, or Division of Muscles. By *Charles H. Rogers-Harrison,* M.R.C.S. Honorary Secretary to the British Medical Association, &c. &c. Illustrated by Drawings. Octavo, pp. 164. London: Churchill, 1842.

Mr. Harrison addresses his work, whose title expresses the principal objects of its author, to the young practitioner and the enlightened general reader.

He sets out by describing the natural structure of the thorax and spine, their bony structure, ligaments, and muscles. From this he passes to the kinds and causes of deviation, on which we have ten chapters. We have gone too far at various times into this subject, to permit us to travel *composito pede* over the ground again. But we may saunter through it, and pick up a flower or some edible root by the way.

Speaking of *slight and temporary deviations,* the result of bad habits, (on which, by the bye, old Monro insisted), Mr. Harrison reprobates go-carts, contrivances for getting rid of children and saving the trouble of teaching them to walk. Children, who cannot stand alone, sustain themselves entirely on the upper rim of these baskets or go-carts by means of their armpits; they are rather more hung up by the armpits than supported by the feet, especially when they are left any length of time, as generally happens. In short all these are execrable contrivances, and perfectly inexcusable where the means for proper nursing are possessed.

After a description of Right Lateral Curvature, Mr. Harrison passes to the examination of its causes. Amongst these, the ever-to-be-execrated stays hold a very prominent place. The profession have uplifted their voices against this abomination in vain. Ladies *will* be in the fashion, *will* look like hour-glasses, or wasps, *will* spoil their shapes, and they must do so. But there is some comfort. Happily Mrs. Mills has introduced elastics, and an improved formation of stays, especially the banishment of upright pieces. Yet still, says Mr. Harrison, "the great evil escapes remedy; *all stay-makers leave the stays sufficiently narrow to admit of being drawn far too tight either for health or beauty.* Parents should see to this: the stay-makers will not, for fear of offending their young customers." We would advise parents to lay Mr. Harrison's counsel to heart. Mr. H. advises that the stays of young girls "be freed as much as possible from the stiff pieces of whalebone, and the steel plates which give them the appearance of a case of instruments," and that they be formed chiefly of a firm, but elastic, tissue, which by giving a slight support to pendent parts, may yield to all motions without injuring the free development of the breast, and without destroying the muscles that regulate the spine. All this is we think indisputable. Certainly this is a common sense and seems a very fair proposal. Whether the ladies will accept it is another matter. Whatever may be the case after parturition, we think there can be little doubt that, prior to pregnancy, the female figure would generally *look* better without formal stays. At all events they should be of the most elastic and least compressive materials.

Mr. Harrison's opinion of the efficient cause of lateral curvature appears to be this, that "in most cases of considerable deviation, the curvatures are chiefly produced by the derangement of the muscles and of the intervertebral ligaments." And he seems to think (a fanciful idea) that the reason why the change is most apt to occur at the period of puberty is—the saturation of the organs with liquid and their softness at that time. It seems more likely that, the epoch of maturity being that of rapid growth is necessarily one of weakness, and the spine having to support great superincumbent weight is naturally a part where such weakness would be felt.

In a chapter on the operation of the muscles in the production of spinal curvature, we find a theory proposed which, whether new or not, is new to us, and is not without ingenuity.

Mr. Harrison, after stating that a fixed point is necessary for the action of muscles, proceeds to argue, that while the spine is straight it is flexible, but if bent it becomes firm by reason of the opposition between the elastic power which tends to straighten it, and the power which curves it. The application of this idea to spinal curvatures we shall quote.

"In the case of movements of the right arm in a weak individual, such as we have supposed, the curvature of the vertebral column to the left will be readily produced, not only by the muscles which directly move it, but still more by the intercostals, the square muscle of the loins, the broad muscle of the back, the inferior portion of the great pectoral, and even a part of the abdominal muscles. This column being once thus curved, all its upper portion will afford a firm point of support to the muscles, which in their turn must fix the shoulder-blade for the motions of the arm. The left shoulder will be much lowered; the arm of this side will even be pressed against the body; and the right shoulder, slightly raised, will be maintained in that state as long as is required for the efforts to be made with the right arm.

"The most common observation bears out this physiological theory of the points of support which the right limb derives from the opposite curvature of the spinal column. The fencing-master on guard, has his spinal column forcibly bent to the left side; the child who attempts to open a door with the right hand, is bent to almost a semi-circle; and the player of tennis, ready to seize the ball which passes with rapidity, waits in that position to secure the precise motion requisite to meet it.

"We must further remark, that the precision and exactness of certain delicate movements require as much the upper portion of the spinal column to be fixed, as certain abrupt and powerful motions.

"A young person embroidering at a frame, drawing or writing, is obliged to guard against the vaccillation of the spinal column, which would produce that of the arm and hand; and consequently, without bending the whole of the vertebral column, as in cases of energetic action, preserves it curved in its upper part however slightly, a circumstance which soon brings on the inverse curvature of the lumbar region.

"We now see that the bending of the spinal column in the superior part of the dorsal region, which is necessarily accompanied by a difference in the height of the shoulders, instead of being an effect of a vicious habit, is no more than an indispensable condition for the execution of certain actions of the right arm, so that if one ask a weak and delicate person to perform these motions, keeping herself quite straight, he requires of her a thing physically impossible; which explains a circumstance so well known, and so frequently met with by practitioners, that it is often quite impossible, in spite of the most frequently repeated care and remonstrances, to obtain from the most docile and submissive young person, that erectness so desired by mothers. We hear daily the most bitter complaints on this subject, and there are no young and delicate girls who have not frequently been accused of obstinacy or of negligence in this respect.

"We may perceive that it is not absolutely indispensable that the curvature of the spinal column should take place on the opposite side to the active limb, in order that the stability of which we have spoken may be secured in the exercise of certain precise and minute movements of the upper extremities. This fixedness would also be produced, although with less advantage, by a flexion of the side of the acting limb; and this may be actually observed when the hand acts upon a point placed on a level with the height of the pelvis.

"There are, however, two reasons why curvatures to the left should be less common: the first is that the opposite curvature offers more advantage to the action of the right limb: the second, that the dorsal region of the vertebral column presents early, in all persons, a slight lateral concavity on the left side. It has been seen that this natural or rather general curvature is to be attributed to the same causes as accidental curvatures, and not, as has been asserted, to the presence of the aorta.

"To sum up these considerations, we conclude that a certain degree of flexion in the upper part of the dorsal region of the spinal column, and more frequently to the right, is with weak patients, an indispensable condition to the stability necessary for the violent motions of the superior extremities, as well as for all motions of the hand requiring exactness and precision, and that the state of curvature, independent of the will of individuals in that situation, must be preserved absolutely during the continuance of these kinds of action."

The extract is a long one, but we could not do justice to the hypothesis by abbreviation. It strikes us that the practical part of the business is plain. Granted that curvature of the spine is a natural attendant on certain muscular actions. That curvature, if persistent or excessive, is a defect, or it may even be disease. The motions, then, that favour its development should be avoided, or they should be neutralised by other motions, or by other means. And this brings us to the every-day practice of combining and regulating muscular exercises in the deformed and in those threatened with deformity.

We think it must be granted that, after all, it is not mere muscular action which causes spinal curvature. Look at the followers of many mechanic arts, exercising muscles very partially—for instance at the fencing-master. Here the spinal muscles may be acting upon one side and the muscles of the shoulder on the other, and yet there is grace, agility, and symmetry.

Distortions are to be looked for in the pale-faced sempstress, the sickly child of the ill-fed artisan, or the delicate victim of education and fashion. The muscles, God knows, are weak enough, but the weakness of the osseous and ligamentous tissues is greater. Fatigue leads to habit—habit to deformity. The curve begun in weakness ends in permanency. Certain muscles may by their action, too, if frequently or long indulged in, affect a skeleton, incompetent to bear its burden, but the fault is really in that skeleton, or rather in that constitutional weakness of which it is only the exponent.

Mr. Harrison devotes a chapter to *Accessory Deviations*, that is, to the Secondary Curvatures consequent upon the first. His explanation of the torsion of the spine which accompanies any important degree of the lateral curvation is possibly correct. He argues that, the erector muscles being fatigued, it becomes necessary to call the accessory muscles into action. As their direction is unfavourable,—

"We are obliged to incline the vertebral column so as to give to the lever for their insertion a shape favourable to their action; and, as the articulating surfaces do not permit the spinal column to bend in a direction completely lateral, the vertebræ perform a rotatory movement from right to left, or from left to right, gliding on one another, which produces the twisting that always accompanies lateral deviations in a way more or less marked."

The transverse process on the side of the convexity is carried backwards, that on the concave side becomes anterior. The torsion of the body may be easily conceived.

"To conceive the cause of this extraordinary mode of derangement, it is necessary to imagine, that in a well-marked curvature of the vertebral column, continuing to sustain the weight of the body, the vertebræ of the middle of that curvature are, in fact, in the same situation as if they were urged by a direct and horizontal force on the side of the concavity, towards that of the convexity: In this impulsion, the body of the verterbra, isolated in its anterior and lateral parts, experiences no resistance: but the articular processes are powerfully restrained by their reciprocal connexion. The transverse processes find, in their articulation with the tuberosity of the ribs, a resistance to their deviation, which would be very weak on the part of an isolated rib, but which becomes considerable by its union with the adjoining ribs."

We are not *quite* satisfied with this explanation, but we do not know that we have any better to offer.

We have a description of the left lateral curvature—of the curvature backwards, observed so commonly in aged country people, particularly in gardeners, and seen, too, in girls who grow quickly and stoop, as in embroidering on very low frames, or in writing at very low tables—of the curvature forwards, generally seen between the 6th and 7th dorsal vertebræ and the third or fourth lumbar. This latter curvature results frequently from the habit which girls have of holding the head very back, to produce arching of the figure—it particularly affects children born of ricketty parents; those who are made to walk very early, little girls who are made to wear stays which compress the stomach without descending over the hips, and girls who limp from deficiency in the length of one leg. It is seen, too, in pregnant women towards the close of pregnancy. The projection into the abdominal cavity might injure free development in pregnancy and give rise to an obliquity. It may even interfere with delivery by reducing the space of the pelvis above the sacro-pubian diameter.

The Treatment of Spinal Curvature is handled by our author at some length. When bad habits have given rise to the deformity, the substitution of directly opposite habits will, in an early stage be beneficial. Mr. Harrison is severe on "the mere mechanical practitioners," and on extension and pressure of the spine. If there is inflammation or change of structure going on, it is mischievous, if there is not, it is "absurd."

Mr. Harrison, among other arguments against extension, urges that the cervical vertebræ, from their anatomical disposition, are more particularly affected by it, whereas the principal deviation is in the dorsal. He urges also that "as soon as the column is withdrawn from the action of the extending powers, it will yield to the active effort of the muscles, which first turned it from its natural straightness, and which will now have so much the more power as the column will be less able to resist." Thus, he says, he has "seen a young girl who, after a treatment of ten months had the column perfectly straight when under extension; but when extension was removed, the trunk sunk again, and the deformity appeared greater than before."

"We have also observed," he says, "after two or even three years of treatment, during which the height of the figure had increased considerably, that the upright position could not be maintained without crutches, and on the removal of these supports, the body sank as much or more than before. The crutches, therefore, became thenceforth indispensable."

These are facts, and by facts this question is to be decided. For, after all, a great deal is to be said on both sides, and we confess that in a fair case of spinal curvature unattended with disease of the vertebræ, or with any particular circumstances to contra-indicate the plan, we see no valid physiological reason against judicious extension. Not that we would argue in favour of its violent or indiscriminate employment. But, combined with rest in the horizontal position, and with appropriate exercises, it appears to us, and we rather think that it has proved, neither objectionable nor useless.

While we express this moderate opinion in favour of cautious extension, we cannot find terms too strong for denouncing the manner in which it has sometimes been carried into operation, more especially in France. If our author is correct, the principle has been inculcated that extension should operate rather by jerks than by a slow and progressive stretching. A more reprehensible doctrine, and more dangerous practice cannot, we think, be conceived, and mischief must inevitably follow its employment. Of this, Mr. Harrison relates one or two striking cases. The recklessness of the means is quite worthy of France in which the cases occurred.

It must be owned that Mr. Harrison is very hard indeed on "mechanical doctors," and it is odd that as Dr. Harrison was one of the most ardent in favour of extension, Mr. Harrison is as vehement against it. We hope this picture is overcharged :—

"But why talk of principle to mechanists and mechanical doctors? Their principle is to make money; they know no other. Such even is their quickness to seize opportunity, that in France, some have had the idea of strengthening the effect of their machines by religious observances : thus, in an institution in the Faubourg St. Germain, known by the name of the Institution Sacré Cœur de Jésus, they treat hump-back by mechanical beds, to which are added faith, hope, charity, and fastings!"

Mr. Harrison touches on the division of muscles in the treatment of spinal curvature. He is feebly opposed to it. For our own parts, we think this muscle-cutting has been a ridiculous and not very creditable mania of the day. There has been something very like humbug mixed up with it, and it is likely to go out in a stink. Whatever reasonableness there may be in dividing a single straight muscle, in a state of morbid contraction, that can never apply to such complicated muscular apparatus as the spinal, with all its thousand fasciculi and more than thousand attachments. The very idea seems to our minds preposterous.

*Rational Treatment* forms the heading and the subject of a Chapter. This consists, according to Mr. Harrison, in watching the figure in early life,—in recommending repose for fatigue and slightly sloping backs for the seats of young ladies—in prescribing healthful games of skipping-rope and so forth—in directing that a bad habit should be counteracted by its contrary—in putting a stop to exercises or pursuits which exercise a bad effect upon the spinal column—in directing gymnastic exercises upon "geometrical data."

First of all, Mr. Harrison insists on a just position of the figure. This he defines to be—"the head raised; the chest expanded; the stomach rather retracted; the back slightly hollowed; the knees well pressed back; the toes turned out, at an angle of not less than 45°; the shoulders kept somewhat back; and the arms hanging easily by the sides, with the elbows turned rather in than out."

Mr. Harrison describes a variety of exercises—comprised in the generic terms extension motions, British Military Club exercise, Indian Club exercise, all described and represented in appropriate and correct costume. Swimming, too, he very properly extols.

For the treatment of dorsal curvature, Mr. Harrison recommends—

1. "Making the patient carry on the arm of the side towards which the body leans, and consequently the side corresponding to the concavity of the curvature, a weight so heavy that not being able to support it long by the mere power of the muscles of the arm, the body may be greatly inclined towards the free side."

2. Raising, with the arm corresponding to the depressed shoulder, a weight suspended to a cord passing over a pulley in the ceiling.

3. Making the hand of the concave side turn a winch with a long handle.

4. Playing at shuttlecock with the left hand.

5. See-saw, the patients standing and holding by two cords fixed to the ceiling.

These exercises are best taken without the stays.

*Treatment of Lumbar Curvature.*—For young men Mr. Harrison advises fencing with the hand corresponding to the concavity of the *dorsal* curvature.

We have, we believe, communicated to our readers the pith and marrow of a book, not deficient in merit, nor even in originality, on a subject that would seem to be absolutely threadbare.

---

THE SURGEON'S VADE MECUM. By *Robert Druitt,* Esq. Second Edition.

We are glad to see that this useful little work has arrived at a second edition. As the author justly observes in the preface, short books have generally one of two faults; they are either rendered short by omitting much that is important, in which case they do harm rather than good; or are so condensed as to become most difficult to read, and hard to understand. Mr. Druitt has certainly done his best to avoid both these errors, and has succeeded in a great degree, still, however, it is impossible altogether to escape the latter of the two faults. In consequence of the small space allotted to each subject, no room can of course be afforded for any reasoning upon any point; the bare facts of the case are stated, and these must be learnt by rote by the student who confines his study of the science of surgery to the perusal of this or any similar single work upon the subject. And this is the great objection to this class of books; many students who are desirous of "passing" with as little trouble to themselves as possible, get by heart the contents of one of these small volumes; they are thus able to repeat, parrot-like, the various symptoms, diagnosis, treatment, of any given complaint, but if asked to give their reasons for what they have stated, they are immediately puzzled; it is so, because it is so put down in "Druitt." In what way, then, is such a book useful? Thus: let the student peruse the best works upon each subject, these he will always be able to procure at the library of the hospital to which he belongs; let him then read this book; the principal points, the main facts will be recalled to his mind in the shortest and most succinct manner; and these facts he will be enabled to understand and to reason upon, from his recollections of the larger and more elaborate treatises from which this book is principally compiled.

In justice to Mr. Druitt, we must say that he has rendered this little volume as complete as possible. About one hundred pages have been added to the practical department of the present edition, which contains almost all the novelties of modern surgery, without however introducing too much of the sentimentalism of the present continental practice. The arrangement of the work is also good, and it contains upwards of fifty wood engravings of a superior description, about one half of which are taken from Mr. Liston's work on Surgery, whilst most of the remainder were, we believe, designed expressly for the work.

---

AN ESSAY ON DIABETES. By *H. Bell,* D.M. P. &c. Translated by *Alfred Markwick,* Member of the Parisian Medical Society. London: Highley, 1842.

The Translator informs us that Dr. Bell has so revised this work on the occasion of his translating it, as to render it at least a new edition. It originally appeared in the Dictionnaire des Etudes Médicales. We cannot pretend to offer a copious account of it, as it is and must necessarily be, in the main, a compilation.

Dr. Bell appears dissatisfied with the ordinary division of Diabetes into *Diabetes Mellitus* and *Diabetes Insipidus.* He prefers distinguishing the various forms by the character of the urine. He admits four species—*Diabetes Mellitus, Diabetes with Fatty Matter, Ureous Diabetes, and Aqueous Diabetes.*

When speaking of the increased specific gravity, so characteristic of diabetes mellitus, Dr. Bell praises Baumes' Hydrometer.

"Although the results obtained by this instrument are not perfectly exact, yet they are sufficiently so for all practical purposes. Every 10th of a degree of this hydrometer corresponds to 733 thousandths of a degree of the decimal scale, so that the first degree = 1007.33 and the 6th is nearly equivalent to 1.044."

It certainly is of the first consequence to ascertain the specific gravity of the urine in diabetes, indeed it should not be neglected in any case of disease of the urinary organs.

M. Bouchardat has found that the sugar of diabetes is the same as that of grapes. The occasionally insipid sugar of diabetes is merely a mixture of diabetic sapid sugar with the other elements of urine, such as lactate of urea, lactate of soda, chlorure of sodium, and extractive matter.

Dr. Bell points out the several means that may be resorted to for the detection of sugar in urine. Fermentation with yeast, by means of which one part of sugar in a thousand parts of urine may be rendered manifest, is a delicate, but may be a fallacious one—the polarization of light, rendered evident by an instrument of Biot's, or by a more simple one of Guerard, is another test, difficult, perhaps, of common application—and finally, the *quantity* of sugar is most easily ascertained, though not with absolute precision, by this method :—"The urine is to be evaporated to dryness, the residue is then treated with alcohol, which dissolves the sugar and all the extractiform substances, soluble in this medium. By evaporating slowly this solution, the sugar crystallizes in small grain like crystals, similar to the sugar of grapes; sometimes, however, an uncrystallizable syrup is obtained, which appears to be owing to the temperature being too elevated during the operation."

The quantity of *urea* would seem, consideratis considerandis, much the same in diabetic as in healthy urine. Generally speaking, it is proportionate to the quantity of nitrogen contained in the food.

*Lithic acid* too may certainly exist in diabetic urine.

Diabetic urine that soon undergoes the vinous fermentation would appear to do so, in consequence of a chyliform fluid which forms in the urine and subsides to its bottom, and acts in the fashion of a ferment.

The presence of *sugar in the blood* of the diabetic is incontestable; the constancy of the occurrence and the amount are undecided.

Dr. Bell, speaking of the *complication* of diabetes, particularly dwells on phthisis pulmonalis. A very common complication it is. Granular disease of the kidney is not a very rare one. Dropsy, generally a consequence of the latter, not unfrequently closes the scene.

On the *diagnosis* of diabetes we need not dwell, although practitioners of no mean character have made great mistakes. The pathology is very unsatisfactory, no lesions being constant, and a dozen being insisted on by as many observers.

The prognosis is, according to Dr. Bell, and our own experience, decidedly unfavourable. He has no great opinion, nor have we, of the reported cures in journals. These are as plenty as blackberries.

"It is, in general, easy to reduce the quantity of urine nearly to what it is in health, to diminish the hunger and thirst within certain limits, and to do away with the moral dejection and physical weakness; the sweet taste in the urine has then disappeared, and the cure is speedily announced. However, such is not the case: the urine contains a notable quantity of insipid sugar, its specific gravity is still considerable, and we very soon see the disease return more severe than ever."

With the foregoing statement we quite agree. The *permanent* cures of diabetes are, we fancy, few indeed. The saccharine matter may disappear without a cure. Febrile affections will often lead to the temporary absence of the sugar—so will active purging, or diarrhœa. And in the last stage of the disease the sugar is not evident. This is particularly the case when the pulmonary symptoms become severe.

Of the Etiology, Nature, and Treatment of Diabetes we need not speak. Unfortunately Dr. Bell has been unable to communicate anything new upon these matters. He examines, only to demolish, the various theories that have been promulgated to explain its nature; and he considers an animal food diet, opium, and the warm bath, the best remedies we are acquainted with—sentiments more just than new.

### *Diabetes with Fatty Matter.*

This next occupies the attention of our author. He quotes cases from various writers. For these we must refer to the work before us, or to *them.* We shall merely extract the following brief directions for distinguishing *chylous urine* from urine rendered turbid by pus, phosphates, or urates.

"Purulent urine, when left to itself, becomes transparent after a time, and deposits a white compact substance, with a well-marked level, which does not mingle with the portion of urine immediately in contact with it. By microscopical examination we are enabled to recognise the presence of characteristic globules of pus. The urine which contains fatty matter does not become transparent when left at rest, neither does it give any deposit, but on its surface is produced a stratum having a creamy aspect; when treated by ether it is rendered completely clear, which is not the case with purulent urine.

"Phosphatic urine deposits by rest a whitish matter, but less compact than that produced by purulent urine. This precipitate is immediately dissolved, and the urine rendered perfectly transparent, by the addition of a small quantity of nitric acid. Moreover, by the aid of the microscope, we recognise the crystalline characters of the phosphate of lime, and of the phosphate of ammonia and magnesia.

"Urine loaded with urates is rendered clear by ebullition, and becomes again turbid on cooling. Nitric acid produces a precipitate of uric acid, easily recognised by the microscope."

Diabetes with fatty matter seems rather less severe than diabetes mellitus; it requires much the same sort of treatment.

Some account is given of diabetes with excess of urea, and diabetes insipidus. But we find on these subjects nothing to detain us. On the whole, this little work contains a very good account of diabetes and deserves attentive perusal.

---

### ON THE DIFFERENT FORMS OF INSANITY, IN RELATION TO JURISPRUDENCE, designed for the use of Persons concerned in Legal Questions regarding Unsoundness of Mind. By *James Cowles Prichard,* M.D. F.R.S. M.R.I.A. &c. &c. 12mo. pp. 243. London: H. Baillière, 219, Regent-street, 1842.

Dr. Prichard informs the reader in an advertisement, that his "design is to convey to persons who either regularly or accidentally are engaged in affairs referring to lunatics, or in trials in which there is question of the sanity or insanity of individuals, such information respecting the different kinds and modifications of mental unsoundness as it may be required for them to possess, in order that they may be enabled to determine on verdicts, or to direct and instruct juries to that effect." He describes the different kinds of madness as they actually exist: that is, as Dr. Prichard thinks that they exist—and he has (we are sorry for it) invented some new names. This may give some idea of the work.

We shall not pursue our author in detail through his Sections and Chapters, but content ourselves with selecting any statement or opinion for comment or for acquiescence, as the case may be.

The first two Sections are occupied with an examination of the opinions, past and present, of English lawyers on the kinds of mental unsoundness. Dr. Prichard shews that the ancient ideas were absolutely erroneous, and those of the day defective. And this leads him to dispute the existence of "monomania," or at all events, to dispute its frequency. Dr. P. observes:—

"It seems, on the whole, to be the settled doctrine of English courts at present, that there cannot be insanity without delusion, or as it is otherwise expressed by physicians, without illusion or hallucination, that is, without some particular erroneous conviction impressed upon the understanding, the affected person being otherwise in possession of the full and undisturbed use of his mental faculties. This is the doctrine of partial insanity, so that a man is supposed to be mad upon one point, and sane on every other particular; a state in itself most incredible. The existence of partial insanity is only admissible in one point of view; namely in that which was taken by the Lord Chancellor (Lyndhurst,) in commenting on the judgement of Sir John Nicholl. 'It is that the mind is unsound, and not unsound on one point only and sound in all other respects, but that this unsoundness manifests itself principally with reference to some particular object or person.' That this is a correct account of the condition of those who labour under what is termed 'partial insanity' will be made, as I trust, abundantly clear in the following pages, in which it will be shown that the disorder is not of that confined and restricted extent of which it is represented to be, and that mental derangement, in almost every case, not only involves a disordered exercise of the intellectual faculties, but extends even farther than the understanding, and implicates more remarkably the moral affections, the temper, the feelings and propensities; that it affects, in reality, the moral character even more decidedly than the understanding."

We confess that our own observation accords in the main with Dr. Prichard's. We do not remember ever to have seen an instance of what could be considered, au pied de la lettre, monomania. There has always, when the patient was narrowly scrutinized, turned up some evidence of eccentricity or perversion of the moral sentiments. At the same time, we can readily conceive, that such a thing as delusion upon one point, independently of any other appreciable derangement, intellectual or moral, *might* occur. To us this does not seem incredible, for it is only an exaggeration of what avowedly happens.

In the next Section Dr. Prichard enumerates the forms of Insanity—mania —moral insanity—monomania—insane impulse, or instinctive madness—fatuity or mental decay—and idiotism, or original weakness.

The description of *Mania* offers nothing for selection. On *Moral Insanity* Dr. Prichard dwells at some length, the term and the original description of the disease having, it would seem, been his own. The subject is one which is now attracting attention, and deserves the serious consideration of the profession. Dr. Prichard thus defines the disorder.

"Moral insanity is a disorder of which the symptoms are only displayed in the state of the feelings, affections, temper, and in the habits and conduct of the individual, or in the exercise of those mental faculties which are termed the active and moral powers of the mind. There is in this disorder no discoverable *illusion* or *hallucination*, or false conviction impressed upon the belief similar to the delusive or erroneous impressions which characterise monomania. It is often very difficult to pronounce with certainty, as to the presence or absence of moral insanity, or to determine whether the appearances which are supposed to indicate its existence do not proceed from natural peculiarity or eccentricity of character. The existence of moral insanity is palpable and easily recognised only in those instances to which it comes on, as it often does, after some strongly marked disorder affecting the brain and the general state of health, such as a slight attack of paralysis, and when it displays a state of mind strikingly different from the previous, and habitual or natural character of the individual. If a person of quiet and sedate temper, little subject to strong emotions, becomes excitable, violent, impetuous, thoughtless, and extravagant to such a degree as to surprise his friends and relatives, a suspicion is often produced that this change may depend upon a disordered state of mind. There are many individuals who are subject to alternate fits of excitement and depression; the contrast renders the peculiarities of such persons apparent. The fact that they are so affected is always known to their families, but they are not suspected of insanity, unless the affection is very strongly marked."

Dr. Prichard, after relating a case and quoting a passage from a former work, continues :—

"In a state like that above described, many persons have continued for years to be sources of apprehension and solicitude to their friends and relatives; the latter, in many instances, cannot bring themselves to admit the real nature of the case. The individual follows the bent of his own inclinations; he is continually engaging in new pursuits, and soon relinquishing them without any other inducement than mere caprice and fickleness. At length the total perversion of his affections, the dislike, and perhaps, even enmity, manifested towards his dearest friends, excite greater alarm. When it happens that the head of a family labours under this ambiguous modification of insanity, it is sometimes thought necessary from prudential motives, and to prevent absolute ruin from thoughtless and absurd extravagance, or from the results of wild projects and speculations, in the pursuit of which the individual has always a plausible reason to offer for his conduct, to make some attempt with a view to take the management of his affairs out of his hands. The laws have made adequate provision for such contingencies, and the endeavour is often unsuccessful. If the matter is brought before a jury, and the individual gives pertinent replies to the questions that are put to him and displays no particular mental illusion—a feature which is commonly looked upon as essential to madness—it is most probable that the suit will be rejected. Persons labouring under this disorder are capable of reasoning or supporting an argument upon any subject within their sphere of knowledge that may be presented to them; and they often display great ingenuity in giving reasons for the eccentricities of their conduct, and in accounting for, and justifying the state of moral feeling under which they appear to exist. In one sense, indeed, their intellectual faculties may be termed unsound; they think and act under the influence of strongly excited feelings, and persons accounted sane are, under such circumstances, proverbially liable to error, both in judgment and conduct."

Dr. Prichard proceeds to shew, that a large proportion of the inmates of lunatic asylums labour under "moral madness" only, and to narrate several interesting cases. These we do not think it requisite to introduce.

The following general remarks comprise the main features of cases of this kind.

1. Great exaltation of animal spirits is the principal feature of many cases.

"Such persons are, sometimes, only more cheerful, lively, active, and restless, than their own natural character or disposition. They are fond of society, talkative, full of schemes and projects, never contented to be alone, or in their own domestic circle; form new attachments, and break them continually, and at the same time have little real affection, especially towards their own relatives. They are thoughtless and extravagant, sometimes generous to excess; would ruin themselves by making presents, or by mere squandering of money. Some make great purchases of ornaments and trinkets."

Others make great purchases in the way of their business. Such a state of excitement is, in many instances, a permanent consequence of mania.

2. In other cases, excitement alternates with periods of gloom and depression. In some, the periods of excitement are separated by long intervals. Many men, in this state, addict themselves to intoxication. Others, from leading a life of respectability and probity, display a total alteration of character; the affectionate and constant husband becomes a debauchee—the man of his word a liar—the quiet man a spendthrift and a blackleg—the conscientious man a hypocrite or scoffer. All this may occur spontaneously—or it may follow an injury of the head —or it may appear in families known for madness in their members. And Dr. Prichard thinks that many of the famous characters in history, Christina of Sweden, Paul of Russia, and Frederick the Second of Prussia, &c. have been mad—a sentiment in which he has been forestalled by the poet who has sung:—

"From Macedonia's madman to the Swede."

But these views were not intended to be, nor could they be barren of practical results. If "moral madness" be madness, it must be treated as such. Then come the writ de lunatico, the jury, deprivation of civil rights, and personal restraint. A serious consequence of what usually passes for errors in conduct, for strong passions, or for vicious sentiments! How then shall this moral madness be defined, how separated from eccentricity, from weakness, from vice, from guilt? Where, on the one hand, shall free agency be curtailed by law, and criminality be screened from punishment, upon the other? To us it is palpable that no words, no distinctions, no fine nor coarse definitions will serve. Whatever pains may be taken, whatever subtlety may be employed, it will be impossible to exclude the free agent of sane mind, but of loose principles, from the press of "moral madness," and, if this were acted on, the world would be one great lunatic asylum.

The fact is that whatever may be said of moral insanity, its presence or absence can only be determined by a consideration of the circumstances of the individual case. If the extravagancies of the individual reach a certain point, the common sense of mankind will decide that it is insanity—if they fall short of it, we cannot with a due regard to the interests of society and to personal freedom, pronounce that the law is justified in interfering. This is the view we have always taken of the matter, and we are glad to perceive that Dr. Prichard is substantially of the same opinion. He says:—

"The question which jurors will have to determine is, not whether the person whose case is under examination is afflicted with insanity according to any abstract definition, or general notion, as to the nature of that disease, but whether his mental state is individually such as to render him unfit to be at large, and to be entrusted with the care of himself and his property. It is important to be aware of the fact, that persons may fall into a state that deprives them of this capability, or of the power and inclination to govern their own conduct with propriety and in consistency with their own habitual principles of action, and that through the effect of disease, without displaying that phenomenon which was heretofore considered as essential to madness, and the sole criterion of its existence; but whether in each particular case, the person afflicted is in such a state as to require legal interference with his personal liberty, and the exercise of civil rights, can only be determined by evidence upon his individual case."

In his Section on *Monomania*, Dr. Prichard reiterates and amplifies the position, to which we have already adverted, *that* the prevalent idea of its nature is incorrect. Rarely, indeed, if ever, do we see a mind unclouded with one single false notion only. The delusion is generally, according to Dr. Prichard, grafted on

" moral insanity." It constitutes, indeed, the most striking feature of the case, but there has been previously a perversion of the moral character, feelings, affections, and habits of the individual.

" The social affections are either obliterated or perverted; some ruling passion seems to have entire possession of the mind, and the hallucination is in harmony with it, and seems to have had its origin in the intense excitement of the predominant feeling; this is always a selfish desire or apprehension, and the illusory ideas relate to the personal state, and circumstances of the individual. A late French writer, M. Leuret, terms monomania, 'le délire des passions;' he has given the details of several cases, which strongly exemplify the preceding remark, and which should be read by those who are desirous of studying the nature of monomania.

" The illusions or false impressions of the monomaniac have always, as I have said, reference to himself. They relate, sometimes, to his fortune, rank, personal identity; at others, to his health of body and his sensations. In the former class of cases, the patient feeling himself unhappy, fancies himself in debt, ruined, betrayed; or, being disposed to an opposite state of feelings, possessed of great wealth and affluence, and superior to all mankind: the difference of these impressions seems to depend upon the different state of spirits; the persons affected by the former kind of impressions are those whose minds are predisposed to gloom and forebodings of ill; the latter kind affect the sanguine and excitable."

Illusions and hallucinations are remarkable traits of monomania. The patient believes that he has actual sensation and perception of what does not really exist. M. Esquirol has proved that these erroneous impressions frequently, at all events, take their rise in morbid bodily sensations. One woman fancied that a regiment of soldiers was fighting in her belly, another that the Apostles and Evangelists were quartered there; both had the intestines agglutinated by chronic inflammation. The sense of hearing is most frequently the seat of the illusion.

Dr. Prichard points out the distinction between hallucinations and illusions.

" M. Leuret terms hallucination a mental phenomenon is intermediate between sensation and conception. It corresponds with sensation in the circumstance, that it occasions, like sensation, the belief of the existence of an external operation on the organs of sense and of an external body which acts upon them. In reality hallucination may be supposed, with probability, to depend upon a morbid state of those operations of the brain which in a healthy condition are connected with simple conception and reverie. These operations, which are momentary changes in the condition of the brain, in the usual and healthy state of the system, take place without giving rise to belief in the existence of any past or present cause or external agent. In consequence of the morbid change such actions of the brain are converted into those which produce real perception and actual memory or recollection, so that the person affected is impressed with the belief that he really sees and feels, or remembers as objects of former sensation, what he only conceives or momentarily fancies. But this phenomenon alone would not constitute in man a lunatic. In general the understanding corrects erroneous perceptions, and if the organs of sense are in such a state as to present unreal objects to the mind, reason convinces the individual of the actual condition under which he labours. Ocular spectra and morbid dreams are thus perceived to be what they really are. Cases are on record which answer to this account, as that of Nicolaï, who for some days previously to his death saw phantoms which he recognised as existing only in his own perceptions. But if a person believes in the reality or more properly in the externality of a phantom or a ghost-scene, it could not constitute him a madman; and this consideration might have been sufficient to convince our predecessors, that their definition of insanity was founded on mistake."

The fact is, (so it seems to us) that the distinction between sanity and insanity is only a question of degree. The hallucination or illusion, call it which you will or be it what it may, reaches a certain point, and we say the man is mad—is short of that, and we deem him sane. A misanthrope who merely believes that the world is made up of cheats and villains may be thought a clever fellow—push his suspicions of human nature a very little farther, and he is insane on the subject of conspiracy or danger. Dr. Johnson believes in the Cock-lane Ghost and he is deemed merely credulous—another man sees visions or dreams dreams, and he is a lunatic. We may readily conceive a community so far advanced in intelligence, as to look upon existing nations with their gross and idle superstitions as no better than Bedlamites.

To return, however, to hallucinations and illusions, we do not perceive that Dr. Prichard has clearly pointed out a distinction between them after all. If it be " illusion" to hear a voice which does not speak, in what does that differ from hallucination, for that " is the belief of the existence of an external operation on the organs of sense and of an external body which acts upon them. ?" If that be not illusion then what is illusion?

On *Melancholia,* and on *Instinctive Madness* or the *Uncontrollable Impulse to perpetrate crimes,* we need not touch; as species of the latter, Dr. Prichard dwells on the *Homicidal Propensity* or *Phonomania,* on the *Propensity to Arson,* on the *Propensity to Suicide,* and on the *Propensity to Theft.*

Dr. Prichard inquires whether the mere fact of suicide can be deemed of itself to be evidence of insanity. He infers that it cannot, and we must say that we agree with him. He argues :—

" That suicide is not always the result of insanity we must infer from the calm and deliberate manner in which it has been perpetrated. This act has frequently been the result of rational motives, which can be well appreciated by sane persons as not altogether devoid of influence, of motives well weighed, of deliberate, calm determination of the will. It results from a desire, not in itself contrary to nature or reason, to escape from anticipated evils, from the sufferings of life protracted under circumstances which promise only shame or misery and disappointment. This is not the character of the acts of a madman, but rather of a person possessed of reason, though under the influence of despair, of passions habitually ill-regulated, and uncontrolled by a sense of duty and religion.

" The frequent practice of suicide in many countries, where it has been looked upon as in no way culpable or indicative of mental disorder, removes it from the class of those anomalous and unaccountable acts depending on insane impulses. By many of the ancients suicide was rather commended. Pliny terms it the greatest privilege that the gods have left in the power of men amid the calamities of human life. At Ceos, the country of Simonides, it is said to have been a popular maxim that every citizen ought to destroy himself when he attained the age of sixty, and it was supposed shameful to survive the period when a man became unable to serve the commonwealth. Among the ancient Romans, as we learn from Tacitus and other writers, suicide was the general result, among the nobles, of falling into misfortune or public disgrace, or under the displeasure of the Emperor. Among the ancients, suicide was thought by no means criminal, and the sentiment of Socrates, who censured it, is recorded as something novel and remarkable. A similar observation may apply to China in the present day, where a Mandarin, or public officer, who falls into disgrace, scarcely thinks of any other termination of his career. In the Chinese plays, translated long ago and published by Duhalde, the unfortunate actors destroy themselves, as if this was a thing to be regularly expected."

He brings forward as other instances of the absence of insanity, cases where a father and mother have agreed to murder their child and destroy themselves, and where lovers have deliberately committed self-destruction.

We must confess that, if suicide is to be held to demonstrate insanity, we know not what are the evidences of sanity. A man, unacquainted, perhaps, with Revelation, or an unbeliever in it, is oppressed with the ills of life, or is disgusted with its pleasures, and voluntarily seeks what he conceives a final termination to them. His reasoning and his act are equally deliberate, and, supposing his premises to be true, cannot be called irrational. Now there are abundance of cases of suicide of this kind, and, in the declining days of Rome, this species of self-destruction obtained throughout the Empire. Nay, it is even possible for a tolerable Christian to commit suicide and to be sane. Misfortunes of the description most intolerable to a sensitive mind oppress or impend over him, he is sensible of the guilt that he incurs, but unable to support the contumely of the world, or the reverse of fortune, he prefers death and throws himself, however, vainly and wickedly, on the mercy of his God.

We are far from contending that the bulk of cases of suicide in these days are of this description. The infinite majority are the result of insanity, of a mind unhinged by its afflictions or dragged down by bodily disease. And sure we are that, for the interests of society and of humanity, it is better to throw, in almost every instance, the cloak of mental aberration over the unhappy act.

Dr. Prichard devotes a section to the consideration—how far insane persons are irresponsible for crime. The difficulty is to decide to the satisfaction of all, what is or what is not insanity, for, were this attainable, there would be no further difference of sentiment.

Perhaps the leading idea amongst jurists is this: *that* a delusion must be proved to exist, and *that* the criminal act must be clearly connected with it and deduced from it. Our ancient statute law has indeed gone far beyond this point and insisted on a complete prostration of reason, as indispensable for a bar to punishment.

The more modern opinion and practice are more merciful as well as more just. Still it must be admitted as medically erroneous, for a man may be insane at the time when he perpetrates a criminal act, although it may be difficult to trace a connexion between it and his delusion, or even to discover a delusion at all. It is "moral madness," the perversion of the moral sentiments and affections, that constitutes the frequent cause of violent and guilty acts, and which may or may not be accompanied by a tangible hallucination.

But if "moral madness" was difficult of determination in cases in which the interests of the individual and his family were alone involved, that difficulty is not diminished where the wider interests of society are implicated. To confine a supposed "moral madman" *might* do him a slight wrong and confer on his family and on society an unquestionable good, for it would remove, at all events, from the sphere of mischief a dangerous or vicious person. But allow this plea of moral madness, so impossible of definition, so hard of discrimination even in the individual case, to form a sufficient and ample defence for the most atrocious acts, and the consequences to society might be destructive. For we must not merely take into consideration the particular crime or the particular individual. The effects of example and of irresponsibility must be looked at, and we must not forget that moral madmen are not altogether so blind to consequences as not to be held in some degree of check by the consciousness that punishment may follow excess. The question is altogether a ticklish one, and we perceive that Dr. Prichard has prudently avoided it.

Each case must still be left to the conflict and caprice of witnesses and counsel, judges and jury. We need not apprehend, at the present time of day, any signal act of inhumanity.

